# Comparing Features for Classification of MEG Responses to Motor Imagery

**DOI:** 10.1371/journal.pone.0168766

**Published:** 2016-12-16

**Authors:** Hanna-Leena Halme, Lauri Parkkonen

**Affiliations:** 1 Department of Neuroscience and Biomedical Engineering (NBE), Aalto University School of Science, Espoo, Finland; 2 Radiology Unit, HUS Medical Imaging Center, Helsinki University Hospital, Helsinki, Finland; 3 Aalto Neuroimaging, MEG Core, Aalto University School of Science, Espoo, Finland; University of Minnesota, UNITED STATES

## Abstract

**Background:**

Motor imagery (MI) with real-time neurofeedback could be a viable approach, e.g., in rehabilitation of cerebral stroke. Magnetoencephalography (MEG) noninvasively measures electric brain activity at high temporal resolution and is well-suited for recording oscillatory brain signals. MI is known to modulate 10- and 20-Hz oscillations in the somatomotor system. In order to provide accurate feedback to the subject, the most relevant MI-related features should be extracted from MEG data. In this study, we evaluated several MEG signal features for discriminating between left- and right-hand MI and between MI and rest.

**Methods:**

MEG was measured from nine healthy participants imagining either left- or right-hand finger tapping according to visual cues. Data preprocessing, feature extraction and classification were performed offline. The evaluated MI-related features were power spectral density (PSD), Morlet wavelets, short-time Fourier transform (STFT), common spatial patterns (CSP), filter-bank common spatial patterns (FBCSP), spatio—spectral decomposition (SSD), and combined SSD+CSP, CSP+PSD, CSP+Morlet, and CSP+STFT. We also compared four classifiers applied to single trials using 5-fold cross-validation for evaluating the classification accuracy and its possible dependence on the classification algorithm. In addition, we estimated the inter-session left-vs-right accuracy for each subject.

**Results:**

The SSD+CSP combination yielded the best accuracy in both left-vs-right (mean 73.7%) and MI-vs-rest (mean 81.3%) classification. CSP+Morlet yielded the best mean accuracy in inter-session left-vs-right classification (mean 69.1%). There were large inter-subject differences in classification accuracy, and the level of the 20-Hz suppression correlated significantly with the subjective MI-vs-rest accuracy. Selection of the classification algorithm had only a minor effect on the results.

**Conclusions:**

We obtained good accuracy in sensor-level decoding of MI from single-trial MEG data. Feature extraction methods utilizing both the spatial and spectral profile of MI-related signals provided the best classification results, suggesting good performance of these methods in an online MEG neurofeedback system.

## 1. Introduction

Motor imagery (MI) augmented with real-time feedback of the modulated brain activity could be an effective method for rehabilitation of motor function in patients who are unable to perform overt limb movements [1–3]. MI, as well as overt motor acts and somatosensory stimulation, is associated with a suppression of 10- and 20-Hz oscillations over the sensorimotor cortex and a rebound of these after the end of MI, motor activity or stimulation [4]. These oscillations are often referred to as the mu rhythm, or more generally as sensorimotor rhythms (SMR). Movement-related suppression and rebound of this rhythm are often referred to as event-related desynchronization (ERD) and event-related synchronization (ERS), respectively. ERD and ERS during overt hand movements were detected in magnetoencephalography (MEG) by Salmelin and Hari [5]. Later, such dynamics has been observed in MEG and electroencephalography (EEG) also during MI [6,7], attempted movements [8], and movement observation [9]. Because the neural activation patterns during MI and executed movements are highly similar [7,10,11], MI-based neurofeedback training could serve as an alternative or ancillary to physical therapy. Regular MI training could facilitate neural plasticity and improve motor skills in e.g. patients suffering from stroke.

The majority of studies on noninvasive MI-based brain—computer interfaces (BCI) have been conducted with EEG. MEG has also been utilized due to its better source localization accuracy compared to EEG [12] and it has already demonstrated its usefulness in neurofeedback [13–15]. Advances in machine-learning and signal-processing algorithms have enabled BCIs which detect users’ intentions by classifying brain activity in real time and by giving feedback according to the classification results [16]. In particular, BCIs based on classification of limb MI have been studied and developed, since they have many potential applications in both neurobehavioral experiments and clinical interventions.

In recent years, there has been rapid development of noninvasive MI-based BCI systems (see a review by He a colleagues [17]). SMR are used as a control signal in many rehabilitative and assistive devices, as they offer the possibility for continuous control of direction, velocity or acceleration of the external device. Other types of control signals, e.g. steady-state visual evoked potentials (SSVEP) or P300, do not readily allow continuous control, and are therefore less popular in movement-related BCI systems. Control of two-dimensional [18] and even three-dimensional [19–21] movement has been achieved with EEG-based BCIs utilizing SMR. However, current classification algorithms could be still improved in terms of accuracy, generalizability and learning time; especially the latter is critical in patient studies as the time needed for calibrating the classifier should be as short as possible. As many different methods have been developed for decoding brain activity, it is important to compare their performance to find out the most effective one. Comparison and further development of the existing decoding algorithms is the main motivation for our research.

Extracting the relevant brain activity from MEG or EEG is the main challenge in the classification of neurophysiological measurements. The number of extracted signal features (e.g. amplitude values, frequency content, spatial patterns) quickly becomes much higher than the available number of single-trial samples corresponding to the task of interest. As most classification algorithms require only a few relevant features for reliable discrimination between classes, it is necessary to reduce the dimensionality of the feature space without losing relevant discriminative information. Dimensionality reduction can be performed with spatial filtering methods, such as Common Spatial Patterns (CSP), which have yielded promising results in discriminating MI patterns in EEG [22–26]. CSP filters project the original signals to components having maximum differences in covariance between two classes. Several extensions, such as Filter Bank CSP (FBCSP, [27]) and Common Spatio—Spectral Pattern (CSSP, [28]), have been suggested for improving the robustness of CSP. Spatio—Spectral Decomposition [29] is a novel spatial filtering method, which maximizes the signal power at a frequency of interest while simultaneously minimizing it at the neighboring frequencies. The method leads to the optimization of signal-to-noise ratio at the selected frequency band and extraction of signal components with a desired spectral profile.

Features extracted by time—frequency analysis are efficient for characterizing changes in oscillatory brain activity, such as those occurring during motor execution and MI [30–32]. With a detailed time—frequency decomposition, one can identify the frequency components that modulate in response to the events of interest and extract those components to be used as discriminative features for classification. To focus on the most informative time—frequency features, both the time window and frequency range of the analysed signal should be chosen appropriately. As multiple neural processes can produce oscillations with similar frequency content, one should also know which brain region to target. In short, sufficient prior knowledge about both the experimental protocol and the neurophysiological phenomena under study is necessary for optimizing feature extraction and classification. Furthermore, in most real-time feedback applications, the total delay of the system should be minimized [33], which rules out computationally expensive signal-processing and classification methods.

Another major problem in designing an MI-BCI is to adapt the classifier to the high inter-subject variability. The most reactive frequency band, the time interval of the most prominent suppression, and the spatial location showing the largest modulation may all differ between subjects and different MI tasks. In addition, the MI-related activation even in the same subject usually varies to some degree between sessions. Despite these sources of variability, the performance of the classifier should ideally remain constant to enable repetitive neurofeedback training over days or weeks. Moreover, an ideal feature set and classifier should generalize across subjects.

In this study, we aimed at selecting optimal feature extraction methods for classifying single-trial sensor-level MEG data of left- and right-hand MI. MI-related feature extraction methods for MEG have been studied previously by Spüler and colleagues [34] who compared functional connectivity features and spectral features for discriminating motor imagery from a mental calculation task. Also, Kang and co-workers [35] compared different CSP methods in the discrimination of left- and right-hand motor imagery in a simultaneous MEG/EEG measurement. In the current study, we compared features extracted by time—frequency decomposition, spatial filtering methods and by the combination of these two. The tested time—frequency methods comprised short-time Fourier transform (STFT), continuous Morlet wavelets (TFR), and multitaper power spectral density (PSD). Spatial feature extraction methods included CSP, FBCSP and SSD, followed by the quantification of band-limited power. In addition, we examined whether using SSD for dimensionality reduction prior to CSP filtering improves the discriminative power of CSP, as suggested in a recent study [36]. Finally, STFT and TFR features were extracted from the CSP-filtered data. Each feature extraction method was evaluated in terms of subsequent classification performance. We also tested four different classification algorithms to verify that in MI decoding feature extraction plays a more important role than the selection of a particular classifier. The factors underlying the differences in the classification accuracy were investigated by estimating the correlation between the mu modulation amplitude and average classification accuracy across subjects.

## 2. Methods

### 2.1. Subjects

Nine healthy volunteers (5 females, 4 males, age 25.8 ± 1.4 yrs, all right handed by self-report) were recruited for this study. None of the subjects reported any neurological illnesses or motor deficits, and all subjects had normal or corrected-to-normal vision. The study was approved by the Aalto University Research Ethics Committee. The research was carried out in accordance with the guidelines of the declaration of Helsinki, and the subjects gave written informed consent prior to the MEG measurements. None of the subjects had previous experience in MI neurofeedback training.

### 2.2. MEG measurements

MEG was recorded with a 306-channel Elekta Neuromag^™^ system (Elekta Oy, Helsinki, Finland) located at the MEG Core of Aalto Neuroimaging, Aalto University. The signals were filtered to 0.1–330 Hz and digitized at the rate of 1 kHz. Four head-position indicator (HPI) coils were attached to the subject's scalp for head position estimation and alignment to a common coordinate frame. The visual cues and feedback (see below) were delivered on a screen located approximately 50 cm in front of the subject's eyes by a projector outside the shielded room. During the recording, the raw MEG data were continuously written in 300-ms segments to a network-transparent ring buffer [33,37] hosted by the MEG acquisition workstation (6-core Intel Xeon CPU at 2.4 GHz, 64-bit CentOS Linux, version 5.3). This buffer was read over a local network connection by another computer (64-bit Ubuntu Linux, version 12.04-LTS), which processed the data in real time using functions implemented in the MNE-Python software [38] and presented the visual stimuli using PsychoPy version 1.83 [39].

### 2.3. Experimental paradigm

The experiment consisted of two sessions, both of which included 40 training trials for calibration of the on-line classifier and 40 trials for testing the classification accuracy. In the beginning of each trial, the subject was presented a realistic image of two hands, pictured from above, on a white background. The subject was instructed to fixate on a small cross at the center of the screen throughout the experiment and not to perform any movements. During the MI task, the subject was instructed by a visual cue (see below for details) to imagine left- or right-hand finger tapping, i.e. touching their thumb with each finger in a sequential manner at a pace of 2–3 touches per second. It was specifically emphasized that the subject should perform kinesthetic instead of visual imagery. Each subject practiced the task for a few minutes outside the magnetically shielded room before the measurements.

Each trial began with a black fixation cross presented in the middle of the screen. After 5 s, the fixation cross turned green to indicate the preparation for the following MI task, and in 2 s a black cue arrow pointing to left or right appeared on the screen for 3 s, prompting for the MI task. Thereafter, the trial structure differed for the training and testing phases as follows:

During the training session, a grasp was shown on the same side as the preceding cue arrow.

During the test trials, visual feedback of the MI classification was presented for 3 s. The feedback was graded with 20 levels between a resting open hand and a fully closed fist, based on a linear transformation of the classification probability, and it was given separately for the left and right hand. Thus, the movement presented in the feedback was different from the imagery tapping movement, whose strength cannot be visualized as a static image of a hand. Yet, we chose finger tapping as the imagery condition, because in pilot studies it elicited stronger mu-rhythm suppression than imagery of a grasping movement. However, the grasping movement was better for the visual feedback since it could be easily graded according to classification probabilities for each hand.

**Fig 1:**
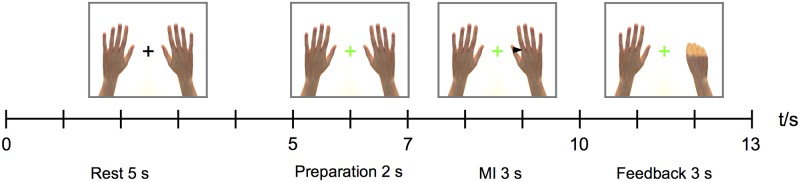
The stimulus paradigm representing the timing and display of the visual stimuli. The 5-s rest period was followed by 2 s of preparation, 3 s of MI of either the left or the right hand, and 3 s of feedback.

The timing and event codes of the stimuli were recorded to the MEG stimulus channel to ensure the correct timing of epochs in the offline data analysis. For an illustration of the experimental protocol, see Fig 1. After the training trials, there was a 30-s break, during which the subjects were shown the instructions for the following test trials. The subjects were allowed to rest eyes closed for 2–5 minutes between the two sessions.

### 2.4. Preprocessing for offline analysis

The MEG data were processed with MaxFilter software (version 2.0, Elekta Oy, Helsinki, Finland) [40], including signal space separation (SSS), coordinate frame alignment to the default head position and orientation and automatic bad-channel detection. Only the signals from the 48 gradiometers above the sensorimotor cortices were included in the data analysis, and the number and locations of the sensors were the same for all subjects; we also tested including all gradiometer channels but that yielded poorer classification results. Magnetometers were excluded due to their lower SNR, and sensors outside the parietal area were excluded in order to reduce data dimensionality and diminish the effect of visual activity, as it has been shown that visual imagery can decrease the classification accuracy in MI tasks [41].

### 2.5. Data analysis

The objective of the off-line data analysis was to improve the online classification accuracy and to optimize the analysis pipeline for further real-time measurements. Several different feature extraction algorithms were compared in terms of their ability to detect relevant features for classification.

#### 2.5.1. Online decoding

For the online feedback, we applied the same, preselected feature extraction method to all subjects; power spectral densities in 8–30 Hz (bins of 1 Hz) were calculated with the multitaper method over the whole 3-s MI epoch and the selected channels. 50 most relevant features determined by chi-square statistics were retained for classification with LDA.

#### 2.5.2. Classifier selection

The most efficient classifier algorithm for offline decoding was selected by comparing the classification accuracies obtained with two commonly used feature extraction methods (CSP and STFT) and four different classifiers implemented in the Scikit-learn software package version 0.17.1 [42]. The efficiency was tested with the left-vs-right classification, since it was considered the most difficult classification task. The classifiers included in this comparison were linear discriminant analysis (LDA), support vector machines with a linear (Linear SVM) and radial basis function (RBF SVM) kernel, and Naïve Bayes (NB). LDA yielded the best accuracy among these classifiers, although the difference was not significant (see Section 3.2). Therefore, LDA was selected for comparing the efficacy of the different features.

#### 2.5.3. Offline decoding

The accuracy for left-vs-right MI and MI-vs-rest classification was evaluated with 5-fold cross-validation over each session; the trials were split into five equally-sized partitions, four of which served as training data to calculate feature transformations, while the fifth partition was used to test the classification performance. The procedure was repeated five times and the resulting classification accuracies were averaged.

In addition, inter-session classification over the two sessions of each subject was calculated; one session served as the training data and the other as testing data, and the classification was performed twice such that both measurements were used once for training and once for testing. The purpose of inter-session classification was to investigate how the variability of MEG signals due to e.g. physiological changes over sessions affects the classification accuracy.

When comparing the effectiveness of different features in classification, the goal is to extract only a sufficient subset of relevant features, i.e. the best possible features from each initial feature space. Therefore, in contrast to many machine-learning studies, we did not use a fixed number of features, but instead tried to find the optimal set of features from all different feature spaces derived from the same data. It is also noteworthy that the initial dimensionality of the feature space varies between different methods, and therefore also the number of relevant features is not constant.

#### 2.5.4. Time—frequency analysis

Time—frequency features were estimated by multitaper PSD, STFT and Morlet wavelets (TFR). Time—frequency features were calculated for the range 8–30 Hz in 1-Hz bins. Prior to STFT, the data were filtered to this frequency range with a finite impulse response (FIR) filter (filter length equal to the epoch length, transition band 0.5 Hz). In the case of STFT and TFR, temporal resolution and thus the number of features was lowered by averaging over the time windows 0.0–1.0 s, 1.0–2.0 s, 2.0–3.0 s and 3.0–4.0 s with respect to the start of the MI epoch. Furthermore, the features were averaged over frequencies, resulting in four average time—frequency features per measurement channel.

#### 2.5.5. Spatial filtering methods

CSP: These transformations were calculated on epochs filtered to 8–30 Hz. The 3 most discriminative spatial filters for each of the two classes, i.e. the 3 first and last columns of the CSP transformation matrix were selected, and the original signals were filtered with them, resulting in 6-component time courses.

FBCSP: The data were filtered to 6 frequency bands of 8–12, 12–16, 16–20, 20–24, 24–28 and 28–32 Hz with FIR filters (filter length equal to the epoch length, transition band 1.0 Hz). CSP transformations and final features were calculated similarly to the basic CSP implementation and separately for all frequency bands. 6 features per each frequency band, i.e. 36 features altogether, were retained. The data dimensionality was further reduced by selecting the 18 best features, according to chi-square statistics, for the final classification.

SSD: This decomposition was performed as described by Nikulin and colleagues [29]. First, the frequency range of maximum mu-rhythm suppression was determined for each subject and session in sensor space using multitaper PSD. Then, the frequency band of interest was set at the frequency showing the largest suppression ±2.0 Hz, and 4-Hz wide side bands below and above the band of interest were set as the flanking frequencies. 20 components in left-vs-right classification and 30 components in MI-vs-rest classification were selected from the covariance matrices for filtering the original data. The number of components was chosen empirically.

SSD + CSP: In this case, SSD was used as a preprocessing method, i.e. for extracting the mu-band oscillatory activity, for noise reduction and for linear decomposition of the data before CSP filtering. We used the same frequency of interest and side bands that were used with SSD alone (see above). The data dimensionality was likewise reduced to 20 components in left-vs-right and 30 components in MI-vs-rest classification. The SSD-transformed signal components were further transformed with CSP, and 3 CSP-filtered components per class were retained.

In all aforementioned spatial filtering methods, the filters were calculated only on the training trials, and the transformations were applied to the test trials. In each case the final feature vector was obtained by squaring the extracted component signals, averaging the samples over the selected time window and taking a logarithm of the average. In order to standardize the features and prevent classifier overfitting, all covariance matrices were regularized with Ledoit—Wolf Shrinkage (LWS) before feature extraction and classification.

#### 2.5.6. CSP + time—frequency analyses

Finally, the time—frequency decompositions were calculated from the CSP-filtered data. Similarly to the previous analyses, 6 CSP components were retained, and the original signals were projected on those components. PSD, STFT and TFR were calculated on the component signals and averaged over 1-s time windows and 1-Hz frequency bins.

#### 2.5.7. Statistical analysis

The statistical differences of feature extraction methods were assessed with Friedman’s test, a nonparametric version of two-way analysis of variance. Since the results of the inter-session classification are independent repetitions of the same test, we used the variant of Friedman test that considers multiple observations per each feature—subject combination; in this case the number of observations was two. In the left-vs-right and MI-vs-rest classification the number of observations was one; since the folds of 5-fold cross-validation are not independent of each other, these classification tasks did not meet the criteria of multiple observations.

In addition, we performed a paired-sample t-test for the results from each fold of the 5-fold cross-validation. The null hypothesis was that the differences between classification results p(i) = p(a)–p(b) were drawn independently from a normal distribution, i.e. method *a* did not yield better results than method *b*. The alternative hypothesis was that better results were obtained with method *a* than with method *b*. The t-test was performed for each pair of methods, separately for left-vs-right and MI-vs-rest classification results.

#### 2.5.8. Level of mu rhythm suppression

The suppression levels of the 10-Hz (actual frequency range 8–12 Hz) and 20-Hz (16–24 Hz) oscillations were calculated for each subject and session in order to examine whether MI-induced suppression was correlated with the subject’s average classification accuracy. Average power was calculated for the baseline (–4.0 ––2.0 s before the start of the MI epoch) and MI (1.0–3.0 s from the start of the MI epoch) for the gradiometers above the sensorimotor cortex. In addition, we calculated the average baseline and MI power for CSP-filtered signals, using the same parameters for CSP as in feature extraction. The power values were averaged over epochs, gradiometers (or CSP components) and frequencies, separately for the 10- and 20-Hz rhythms, resulting in average power values during baseline and MI periods. The suppression percentage was then calculated as [4]:

$$suppression\;=\;\left[\left(power_{MI}\;–\;power_{rest}\right)\;/\;power_{rest}\right]\;\cdot \;100\;\%$$

Pearson’s correlation between 1) 10-Hz suppression and left-vs-right accuracy, 2) 10-Hz suppression and MI-vs-rest accuracy, 3) 20-Hz suppression and left-vs-right accuracy, and 4) 20-Hz suppression and MI-vs-rest accuracy were calculated. We also estimated the correlation between MI-vs-rest cross-validated accuracy and the 10/20-Hz power at rest. In addition, we computed the suppression percentage and correlations between classification accuracies and suppression individually for each gradiometer, and visualized the single CSP component showing the highest suppression for each subject.

### 2.6. Data availability

The data used in the current analyses are available from the authors upon request.

## 3. Results

### 3.1. MI-induced MEG signals

**Fig 2:**
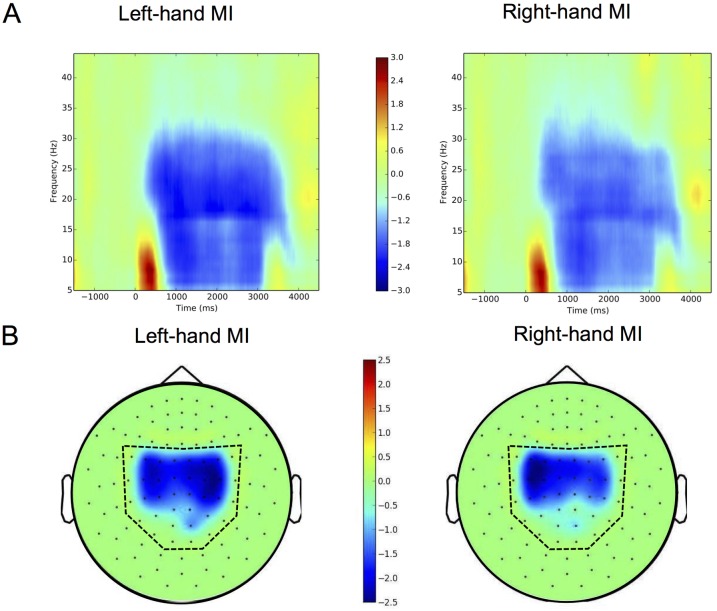
MI-induced MEG signals averaged over subjects. (A) Time—frequency maps for left- and right-hand MI, representing the Z-score with respect to baseline (–2.0–0.0 s from the cue onset), averaged over subjects, sessions, epochs and parietal gradiometers. (B) Corresponding topographic maps, representing the average Z-score over subjects, sessions, epochs and time window of 1.0–4.0 s from the cue onset. The set of 48 planar gradiometers included in the analysis is indicated by a dashed line.

In order to illustrate the MI-induced mu-rhythm suppression and rebound, we calculated time—frequency representations (TFR) over the time course of the MI epoch and the frequency range 5–45 Hz. The TFRs were averaged over subjects, sessions and epochs, and converted to Z-scores: Z = (x– μ)/σ, where μ is the mean and σ the standard deviation of the TFR values. Mean and standard deviation were calculated separately for each 1 Hz frequency band in 0.5 s time windows. The resulting grand-average TFRs for left- and right-hand MI (averaged over channels) and corresponding spatial patterns (averaged over time) are shown in Fig 2. As MI is cued to begin at 0 s, there is an initial power increase at frequencies below 15 Hz, probably reflecting preparation for the following MI. The suppression (ERD) begins at around 0.5 s and changes to a rebound (ERS) after 3.0 s as MI ends. The 0.5-s delay in the beginning of MI is due to the reaction time.

### 3.2. Online left-vs-right classification accuracy

The online classification was calculated for the gradiometer data. Left-vs-right accuracy of the online feedback was fairly low; several subjects remained at the chance level (50%), and the average accuracy over subjects was 62.4%. The results for online classification are summarized in Table 1.

**Table 1:** Online left-vs-right MI classification accuracies for each subject and session. Multitaper PSD features and an LDA classifier were used in the online implementation.

1A	1B	2A	2B	3A	4A	4B	5A	5B	6A	6B	7A	7B	8A	8B	9A	9B	MEAN
48,8	58,5	67,7	61,0	64,4	48,8	56,1	92,7	82,9	51,2	78,1	51,2	41,5	70,7	63,4	58,5	65,9	**62,4**

### 3.3. Offline classifier comparison

The accuracies obtained with the four compared classifiers are presented in Table 2. The average classification over subjects and two feature extraction methods (CSP and STFT) was calculated. LDA yielded an average accuracy of 68.60% appearing superior to the other classifiers. However, the difference was not significant.

**Table 2:** Mean left-vs-right MI accuracy obtained using CSP and STFT for feature extraction and four different classifiers.

Classifier	CSP	STFT	MEAN
**SVM, RBF kernel**	69,56	65,23	**67,39**
**SVM, linear kernel**	70,39	65,46	**67,92**
**Naive Bayes**	67,42	61,70	**64,56**
**LDA**	70,07	67,13	**68,60**

### 3.4. Offline cross-validated classification accuracies

The average cross-validated single-trial accuracy over all methods and subjects was 68.7% (range 35.7–91.4%) in left-vs-right classification and 77.7% (range 49.3–99.4%) in MI-vs-rest classification. Applying CSP filtering before time—frequency decomposition improved the mean accuracies of all time—frequency feature extraction methods.

SSD+CSP combination yielded the best accuracies in both left-vs-right (mean 73.7%, range 59.3–90.2%) and MI-vs-rest (mean 81.3%, range 66.5–99.4%) classification.

Tables 3 and 4 show the mean accuracies of the left-vs-right classification and MI-vs-rest classification, respectively.

**Table 3:** Left vs. right hand 5-fold cross-validation accuracy for each subject and session. The best accuracy for each session is in bold.

SUBJECT/ SESSION	STFT	Morlet	PSD	FBCSP	SSD	CSP	SSD+CSP	CSP+STFT	CSP+Morlet	CSP+PSD	SUBJECT MEAN
**1A**	75,3	70,3	69,2	76,6	70,4	82,7	**82,8**	80,4	81,5	79,0	**76,8**
**1B**	75,2	71,5	74,0	**76,5**	60,3	75,1	65,2	74,0	70,4	70,4	**71,3**
**2A**	59,4	63,4	**70,2**	54,1	66,4	66,3	67,5	59,4	65,0	52,9	**62,5**
**2B**	70,4	**79,0**	69,2	67,7	51,8	65,2	66,5	66,5	70,4	66,6	**67,3**
**3A**	69,0	74,1	73,8	68,6	62,9	59,6	**77,9**	57,4	58,4	59,4	**66,1**
**4A**	76,5	75,2	**79,0**	72,9	60,4	75,1	76,8	70,4	71,6	67,9	**72,6**
**4B**	58,1	63,0	56,8	64,1	58,0	79,0	76,5	76,5	**79,1**	72,8	**68,4**
**5A**	80,2	77,7	68,0	77,6	**81,5**	69,3	80,2	75,3	77,9	74,1	**76,2**
**5B**	79,0	82,9	77,9	74,2	82,9	86,6	**90,2**	89,0	84,1	77,9	**82,5**
**6A**	66,6	67,9	71,8	65,7	56,6	67,7	75,2	74,3	71,5	**75,3**	**69,3**
**6B**	86,5	**91,4**	**91,4**	86,4	59,4	84,0	90,2	87,7	88,9	86,5	**85,2**
**7A**	59,3	65,6	62,9	57,1	65,5	65,4	**66,6**	63,1	61,9	61,8	**62,9**
**7B**	49,2	59,0	56,5	56,5	35,7	61,8	59,3	65,4	65,5	**65,6**	**57,5**
**8A**	55,4	64,0	66,5	57,9	45,5	60,4	65,4	65,4	62,9	**67,9**	**61,1**
**8B**	64,6	61,9	61,8	60,7	61,6	57,0	**65,7**	**65,7**	60,7	64,6	**62,4**
**9A**	66,8	59,3	61,7	65,4	53,1	69,0	76,5	76,6	73,0	**79,0**	**68,0**
**9B**	49,4	55,4	59,2	44,4	65,4	53,3	**70,4**	64,3	63,0	55,6	**58,0**
**MEAN**	**67,1**	**69,5**	**68,8**	**66,3**	**61,0**	**69,3**	**73,7**	**71,3**	**70,9**	**69,3**	**68,7**

**Table 4:** MI-vs rest 5-fold cross-validation accuracy for each subject and session. The best accuracy for each session is in bold.

SUBJECT/ SESSION	STFT	Morlet	PSD	FBCSP	SSD	CSP	SSD+CSP	CSP+STFT	CSP+Morlet	CSP+PSD	SUBJECT MEAN
**1A**	74,0	78,9	79,5	**97,5**	82,9	95,1	94,4	95,7	94,4	93,8	**88,6**
**1B**	83,8	86,3	80,7	**99,4**	80,2	98,2	**99,4**	95,7	96,3	92,6	**91,3**
**2A**	71,2	69,2	**73,8**	70,0	70,4	73,2	73,1	72,5	73,2	**73,8**	**72,0**
**2B**	72,1	71,8	72,3	61,5	64,3	71,5	**75,2**	67,1	67,1	67,1	**69,0**
**3A**	85,5	86,5	**87,5**	70,2	81,5	76,9	79,3	86,1	86,5	80,8	**82,1**
**4A**	78,9	80,2	80,2	83,2	72,9	**88,2**	85,1	85,7	87,6	87,0	**82,9**
**4B**	82,6	80,7	85,7	76,3	53,0	**87,5**	85,7	82,0	83,2	82,6	**79,9**
**5A**	67,7	70,2	71,4	73,3	53,0	70,2	68,3	72,1	**76,4**	73,3	**69,6**
**5B**	63,9	67,0	63,3	**69,6**	67,8	67,8	66,5	63,4	65,2	66,5	**66,1**
**6A**	91,3	91,3	88,8	**93,2**	87,7	90,7	**93,2**	88,8	90,7	87,6	**90,3**
**6B**	78,2	85,7	80,7	**88,2**	85,2	83,2	85,1	84,5	86,3	78,2	**83,5**
**7A**	71,5	72,7	73,9	78,3	64,1	85,1	79,5	85,1	**86,4**	85,1	**78,2**
**7B**	70,2	77,6	74,5	79,5	70,3	84,4	83,2	86,9	**87,5**	78,2	**79,2**
**8A**	73,9	75,2	73,9	68,9	79,2	65,2	**80,8**	63,9	67,6	64,5	**71,3**
**8B**	85,7	86,3	84,5	83,3	82,7	74,5	**88,2**	71,4	70,7	65,8	**79,3**
**9A**	60,5	**70,1**	68,3	64,6	49,3	58,4	69,5	57,2	62,1	59,0	**61,9**
**9B**	80,0	80,8	**83,2**	63,3	62,9	67,6	75,1	77,0	77,6	74,5	**74,2**
**MEAN**	**75,9**	**78,3**	**77,8**	**77,7**	**71,0**	**78,7**	**81,3**	**78,5**	**79,9**	**77,1**	**77,6**

According to the Friedman test, there was a statistically significant difference between the classification results obtained using different features, both in left-vs-right (χ^2^ = 25.48, *p* = 0.0025) and MI-vs-rest classification (χ^2^ = 22.39, *p* = 0.0077). Post-hoc analysis was conducted using Bonferroni correction for multiple comparisons, implemented in MATLAB (R2015a; Mathworks Inc., MA, USA) function *multcompare*. In the case of left-vs-right classification, there were significant differences between the mean ranks of SSD and SSD+CSP (*p* = 0.0018) and FBCSP and SSD+CSP (*p* = 0.0029). Other pairwise comparisons of the methods did not yield significant differences. In case of MI-vs-rest classification, a significant difference was found between the results of SSD and SSD+CSP (*p* = 0.0113) and between SSD and CSP+Morlet (*p* = 0.0156).

The p-values from paired-samples t-test for the cross-validation folds are shown in Table 5 (left-vs-right) and Table 6 (MI-vs-rest). The results represent the significance level for the hypothesis that the method on each row is better than the methods on the columns. According to these results, CSP+STFT and CSP+Morlet were significantly better than most of the other methods in left-vs-right classification. SSD+CSP yielded significantly better results compared to SSD, but not to any other methods, probably due to the high variance in the folds of cross-validation. On the other hand, SSD+CSP was significantly better compared to all other methods on MI-vs-rest classification.

**Table 5:** Statistical tests (*p*-values) of the differences of the tested methods (first column vs. other columns) in the left-vs-right classification (5 cross-validation folds). Significant (*p* < 0.05) values are in bold.

	**STFT**	**Morlet**	**PSD**	**FBCSP**	**SSD**	**CSP**	**SSD+ CSP**	**CSP+ STFT**	**CSP+ Morlet**	**CSP+ PSD**
**STFT**		0.973	0.916	0.180	**0.003**	0.896	0.350	0.991	0.990	0.875
**Morlet**	0.027		0.315	**0.016**	**0.000**	0.503	0.085	0.906	0.900	0.499
**PSD**	0.084	0.685		**0.038**	**0.000**	0.613	0.121	0.937	0.921	0.602
**FBCSP**	0.820	0.984	0.962		**0.021**	0.988	0.638	1.000	1.000	0.991
**SSD**	0.997	1.000	1.000	0.979		1.000	0.996	1.000	1.000	1.000
**CSP**	0.104	0.497	0.387	**0.012**	**0.000**		**0.041**	0.966	0.956	0.495
**SSD+CSP**	0.650	0.915	0.879	0.362	**0.004**	0.959		0.997	0.995	0.943
**CSP+STFT**	**0.009**	0.094	0.063	**0.000**	**0.000**	**0.034**	**0.003**		0.403	**0.016**
**CSP+Morlet**	**0.010**	0.100	0.079	**0.000**	**0.000**	**0.044**	**0.005**	0.597		**0.015**
**CSP+PSD**	0.125	0.501	0.398	**0.009**	**0.000**	0.505	0.057	0.984	0.985	

**Table 6:** Statistical test (*p*-values) of the differences of the tested methods (first column vs. other columns) in the MI-vs-rest classification (5 cross-validation folds). Significant (*p* < 0.05) values in bold.

	**STFT**	**Morlet**	**PSD**	**FBCSP**	**SSD**	**CSP**	**SSD+ CSP**	**CSP+ STFT**	**CSP+ Morlet**	**CSP+ PSD**
**STFT**		0.993	0.860	0.487	**0.000**	0.961	1.000	0.910	0.984	0.615
**Morlet**	**0.007**		0.099	0.156	**0.000**	0.789	0.999	0.610	0.889	0.200
**PSD**	0.140	0.901		0.304	**0.000**	0.926	1.000	0.824	0.971	0.392
**FBCSP**	0.513	0.844	0.696		**0.001**	0.982	1.000	0.923	0.993	0.640
**SSD**	1.000	1.000	1.000	0.999		1.000	1.000	1.000	1.000	1.000
**CSP**	**0.039**	0.211	0.074	**0.018**	**0.000**		0.991	0.145	0.765	**0.001**
**SSD+CSP**	**0.000**	**0.001**	**0.000**	**0.000**	**0.000**	**0.009**		**0.001**	**0.044**	**0.000**
**CSP+STFT**	0.090	0.390	0.176	0.077	**0.000**	0.855	0.999		0.997	**0.008**
**CSP+Morlet**	**0.016**	0.111	**0.029**	**0.007**	**0.000**	0.235	0.956	**0.003**		**0.000**
**CSP+PSD**	0.385	0.800	0.608	0.360	**0.000**	0.999	1.000	0.992	1.000	

### 3.5. Inter-session accuracy

CSP+Morlet yielded the best results in inter-session left-vs-right MI classification (mean 69.1%, range 50.0–85.2%), and the average accuracy over all methods and subjects was 65.4%. Table 7 represents the results of the inter-session classification. The session indicated in the first column of the table was used as the training data and the other session for the same subject as testing data. Subject 3 had only one session recorded due to technical problems and was thus excluded from this analysis.

**Table 7:** Inter-session accuracy for each subject. The indicated session was used as the training data. The best accuracy for each session is in bold.

SUBJECT/ SESSION	STFT	Morlet	PSD	FBCSP	SSD	CSP	SSD+CSP	CSP+STFT	CSP+Morlet	CSP+PSD	SUBJECT MEAN
**1A**	60,5	56,8	58,0	54,3	64,2	**71,6**	69,1	69,1	**71,6**	69,1	**62,2**
**1B**	69,1	71,6	65,4	61,7	69,1	74,1	72,8	71,6	69,1	**79,0**	**64,7**
**2A**	52,6	**55,1**	52,6	53,9	44,9	50,0	50,0	50,0	50,0	50,0	**52,7**
**2B**	54,6	58,4	54,6	**59,7**	35,1	55,8	55,8	55,8	55,8	55,8	**55,4**
**4A**	67,9	74,1	77,8	72,8	58,0	**81,5**	75,3	79,0	**81,5**	**81,5**	**73,3**
**4B**	70,4	**75,3**	72,8	59,3	56,8	69,1	69,1	72,8	**75,3**	66,7	**66,8**
**5A**	81,5	80,3	82,7	71,6	82,7	82,7	**84,0**	**84,0**	81,5	77,8	**79,5**
**5B**	81,5	84,0	85,2	**91,4**	82,7	82,7	80,2	82,7	85,2	81,5	**81,8**
**6A**	85,2	**86,4**	84,0	84,0	59,3	70,4	82,7	80,3	81,5	72,8	**71,0**
**6B**	66,7	66,7	71,6	**81,5**	69,1	70,4	66,7	65,4	67,9	64,2	**70,1**
**7A**	56,8	**67,9**	61,7	58,0	56,8	58,0	64,2	63,0	56,8	58,0	**56,9**
**7B**	58,0	64,2	58,0	63,0	44,4	61,7	56,8	63,0	**66,7**	55,6	**59,6**
**8A**	63,0	**64,2**	63,0	54,3	55,6	58,0	**64,2**	54,3	55,6	51,9	**61,3**
**8B**	55,6	60,5	60,5	60,5	55,6	**64,2**	**64,2**	61,7	63,0	59,3	**59,3**
**9A**	66,7	69,1	65,4	71,6	63,0	60,5	**76,5**	64,2	65,4	63,0	**63,4**
**9B**	65,4	64,2	69,1	69,1	75,3	75,3	72,8	70,4	**79,0**	75,3	**68,7**
**MEAN**	**66,0**	**68,7**	**67,7**	**66,7**	**60,8**	**67,9**	**69,0**	**68,0**	**69,1**	**66,3**	**65,4**

According to the Friedman test, there was a statistically significant difference between the results obtained using different features (χ^2^ = 20.79, *p* = 0.014). However, the post-hoc test conducted with Bonferroni correction did not reveal any significant differences between the methods.

### 3.6. The effect of individual mu-rhythm characteristics on classification accuracy

We investigated how 1) 10- and 20-Hz amplitudes at rest, 2) the suppression of 10- and 20-Hz rhythms during MI were related to the subject’s average MI classification accuracy. The correlation was calculated both on gradiometer and CSP-filtered data.

For the data averaged over parietal gradiometers, no correlation was found between left-vs-right mean accuracy and suppression of sensorimotor rhythm during MI (for 10 Hz, *r* = 0.143, *p* = 0.584; for 20 Hz, *r* = 0.324, *p* = 0.204). MI-vs-rest mean accuracy was significantly correlated with the 20-Hz suppression amplitude (*r* = 0.510, *p* = 0.037) but not with the 10-Hz suppression (*r* = –0.088, *p* = 0.737). The level of sensorimotor rhythms during baseline was not correlated with MI-vs-rest mean accuracy (for 10 Hz, *r* = –0.260, *p* = 0.314; for 20 Hz, *r* = 0.123, *p* = 0.639). The results were very similar for the CSP-filtered data averaged over components. Left-vs-right mean accuracy did not correlate with suppression of SMR (for 10 Hz, *r* = 0.182, *p* = 0.484; for 20 Hz, *r* = 0.326, *p* = 0.202), nor did SMR power at rest (for 10 Hz, *r* = –0.351, *p* = 0.168; for 20 Hz, *r* = –0.200, *p* = 0.441). Also in this case MI-vs-rest mean accuracy was significantly correlated with the 20-Hz suppression amplitude (*r* = 0.582, *p* = 0.014) but not with the 10-Hz suppression (*r* = 0.008, *p* = 0.977).

**Fig 3:**
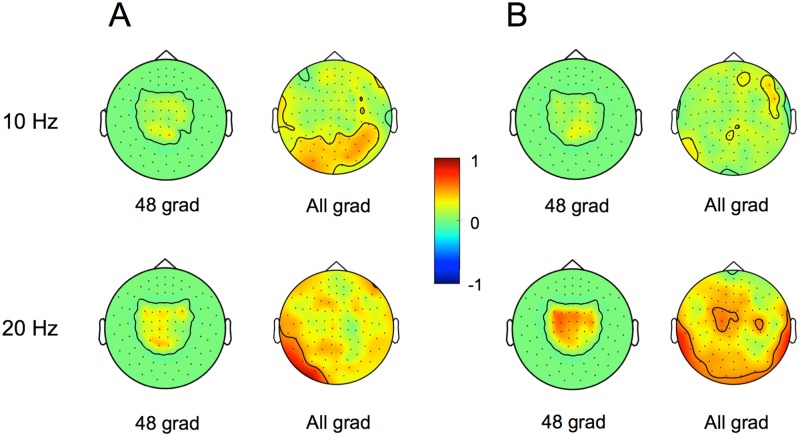
Correlations of classification performance and measures of 10/20-Hz activity at each gradiometer. Correlations between (A) left-vs-right and (B) MI-vs-rest cross-validated accuracy and 10/20-Hz suppression level at 48 parietal gradiometers (left) and all gradiometers (right). In the case of 48 gradiometers, the black contour lines show the outlines of non-zero correlation values.

**Fig 4:**
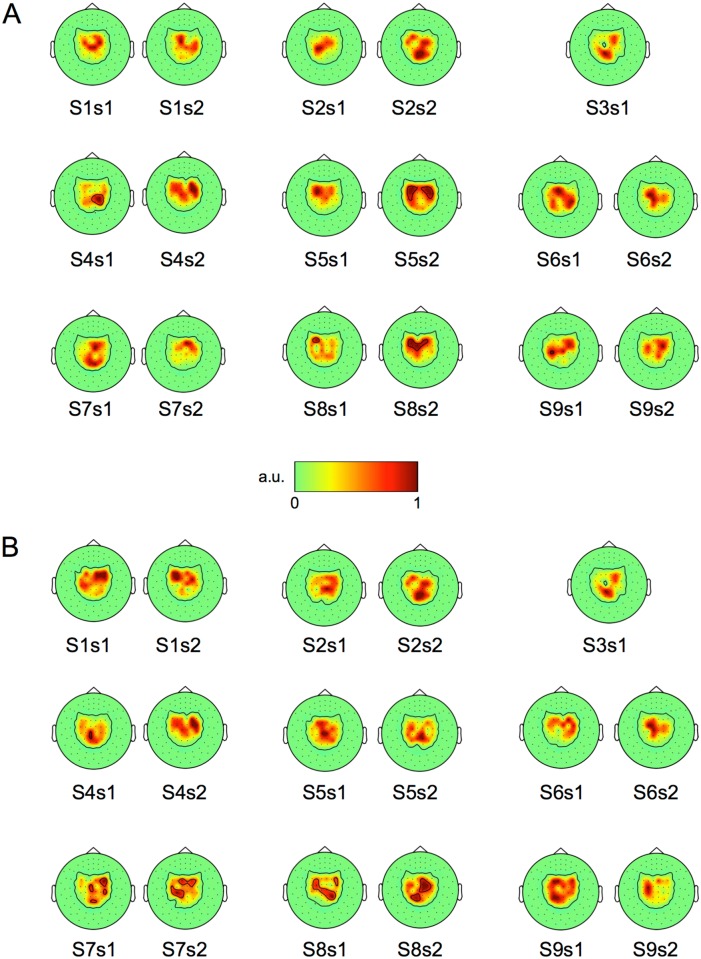
Spatial patterns of the CSP components showing the highest 10/20-Hz suppression for each subject (S) and session (s). Components showing the highest (A) alpha-band and (B) beta-band suppression during MI. The scale is normalized to 0–1 (arbitrary units) for clarity. The outermost black contour lines show the outline of non-zero values.

Correlations were also calculated separately for each gradiometer included in the analyses as well as for each CSP-filtered component. Fig 3 illustrates the correlations between mu-rhythm suppression at each gradiometer and average classification accuracy in left-vs-right and MI-vs-rest classification tasks. The level of beta-band suppression over the sensorimotor cortex shows high correlation with both left-vs-right and MI-vs-rest accuracy. Alpha-band suppression in occipital areas has a fairly high correlation with left-vs-right accuracy. Fig 4 shows, for each subject and session, the spatial pattern of the CSP component showing the highest mu-rhythm suppression in alpha and beta bands.

## 4. Discussion

The main objective of this study was to validate optimal feature extraction methods for characterizing single-trial hand MI in the context of an MEG-based neurofeedback system. Multiple spatial filtering and time—frequency analysis methods, commonly used for feature extraction from EEG/MEG signals, were evaluated in terms of their ability to capture relevant features for subsequent classification.

### 4.1. Evaluation of feature extraction methods

According to our results, spatial filtering methods in general outperformed time—frequency methods in all single-trial classification tasks. In addition, time—frequency features extracted from CSP-filtered signals yielded better classification than the corresponding time—frequency features extracted from gradiometer signals. Among the evaluated methods, the best was the combination of SSD and CSP, which outperformed other decomposition methods (SSD, CSP and Filter-Bank CSP) in both left-vs-right and MI-vs-rest classification. CSP has been found efficient for discriminating single-trial MI patterns [22–24], and our results agreed with these findings, since already the basic CSP yielded good accuracies in the current study. However, adding SSD filtering prior to CSP further improved the classification. As has been shown by Blankertz and colleagues [24], CSP is the optimal method for the extraction of variance differences between two classes, and a prior linear decomposition does not add any new information to that. The increase in classifier performance was most likely due to decreased overfitting compared to CSP alone [36]. The superior performance of SSD+CSP on both left-vs-right and MI-vs-rest indicates the flexibility of the method in discriminating the relevant oscillatory activity from noise. Another advantage of spatial filtering with linear decomposition is low computational cost: the computation time of the proposed methods was negligible (below 0.5 s per epoch for all methods) and thus they are suitable for a real-time interface. In the real-time setup, additional time could be saved if the filters were fitted to the training data only in the beginning of the experiment.

### 4.2. Accuracies in different classification tasks

As expected, the discrimination between baseline and MI yielded better accuracy than left-vs-right-hand MI classification. Regarding the possible application of these methods in neurofeedback rehabilitation, the classification between baseline and MI might be as important as left-vs-right classification. Many stroke patients with motor disabilities have difficulties in mu rhythm modulation in the affected hemisphere. Instead, the unaffected hemisphere shows increased motor-related activity due to reduced intracortical inhibition, as shown during voluntary movements [43] and using paired-pulse transcranial magnetic stimulation [44]. Thus, in the early phase of rehabilitation it might be more useful to target discriminating rest and MI, and once the patients are capable of producing a detectable mu-rhythm suppression during MI of the affected limb, the training could focus on differentiating right- and left-side MI.

Despite the good results obtained with healthy subjects, it is yet impossible to predict the performance of the presented methods in patients. Sensorimotor brain activity is often disrupted and variable in patients with a stroke in the territory of middle cerebral artery. Therefore, training the classifier with individual MEG/EEG data might not be the optimal approach. Tangwiriyasakul and colleagues [45] reported that using motor execution -related EEG signals from the unaffected hemisphere of acute hemiparetic stroke patients was sufficient for training a classifier that differentiated rest from MI. One could also train the classifier with a large database of healthy subjects’ MI-related signals. In this case, the neurofeedback-assisted rehabilitation could be initiated without first collecting the individual (likely low-quality) training data from each patient. However, decoding one subject’s brain activity using data transferred from another subject is challenging and requires additional regularization of the spatial filters [46]. Yet another plausible solution for training the classifier might be to use data collected during passive movements, as suggested by Kaiser and co-workers [47], or during functional electrical stimulation of the target muscles [48].

The within-session left-vs-right accuracies were higher than the inter-session classification accuracies, indicating that there was some variation in the measured brain responses between the two sessions of the same subject. Spatial filtering methods involving CSP performed well also in this situation, yielding above-chance decoding accuracies for most subjects. These findings suggest that spatial filtering increases the robustness of MI classification also over sessions. It should be noted that LWS regularization of the covariance matrix was applied to all spatial filters, as such regularization is known to reduce classifier overfitting.

### 4.3. Inter-subject differences

Classification accuracies varied substantially across the subjects, even though all of them were naïve to neurofeedback and BCI training. The best subjects achieved > 90% left-vs-right accuracy with several different feature extraction methods, whereas the least successful subjects hardly performed over chance level (50%). A likely reason for the low accuracies is the relatively low number of training trials. Previous studies reporting excellent MI classification accuracies have used both highly trained subjects and a large number of trials for both training and testing. For example, the MEG/EEG datasets of Berlin Brain—Computer Interface Competition [49] include hundreds of MI trials recorded from trained subjects. Foldes and colleagues [50] reported that only five minutes of training data are sufficient for training an MEG-based BCI, but they used overt movements instead of MI, and the discrimination was done only between rest and movement. However, it is noteworthy that in our study up to 91% left-vs-right MI classification accuracies were achieved despite the small amount of data and untrained subjects.

We assumed that the inter-subject differences in classification performance could be explained by the differences in the suppression of mu rhythm during MI. As expected, we found that the level of the 20-Hz suppression correlated significantly with the subject’s average accuracy of MI-vs-rest classification. This correlation was slightly stronger on the left hemisphere (Fig 3), probably due to the fact that all subjects were right-handed. In contrast, the left-vs-right classification accuracy could not be explained by the level of mu rhythm suppression. A possible reason for this finding is that the spatial patterns could be more relevant in discriminating right- and left-hand MI, and these patterns varied significantly between subjects. This variation is illustrated in Fig 4, which represents the CSP components with the highest alpha- and beta-band suppression: the spatial patterns tend to be more similar between sessions than between subjects.

In contrast to the results of Blankertz and colleagues [51], we did not find a significant correlation between mu rhythm baseline amplitude and classification accuracy. This finding is probably due to the fact that in our study the baseline amplitude was measured during the short 5-s rest periods between the MI epochs, whereas Blankertz and colleagues collected the baseline data in a separate longer measurement during which the subjects were resting eyes closed. Although in our study the subjects were instructed to perform MI only during the cued periods, it is possible that movement-related activity was present also during the rest periods.

It has been reported that some subjects are BCI illiterate, i.e. not able to operate an MI-BCI despite excessive training [51,52], and our results suggest that insufficient mu rhythm suppression can partially explain this finding. Guger et al. [53] reported that 93% of untrained subjects achieved over 60% left-vs-right MI accuracy after two sessions of training, and 7% of subjects remained below 60% performance. Our off-line cross-validated accuracies are not directly comparable to the aforementioned results, but it is noteworthy that all nine subjects achieved higher than 59% cross-validated left-vs-right accuracy with SSD+CSP features. It is also likely that the subjects can improve mu-rhythm modulation, and thus the classification accuracy, with repetitive BCI training. It would be interesting to examine whether the least successful subjects are able to achieve sufficient control over the MI-based BCI after several training sessions. On the other hand, unsuccessful subjects could be excluded from further studies by measuring suppression of sensorimotor rhythms in an initial screening session.

### 4.4. Limitations and future studies

There were certain limitations in the current study that should be addressed before we can design a real-time MEG neurofeedback system feasible for e.g. rehabilitation. First, we only used visual stimuli and feedback in our experimental paradigm. However, proprioceptive feedback could enhance the subjective feeling of successful motor imagery and thus be a more natural feedback modality for neurofeedback learning [54,55], especially during rehabilitation of motor functions [56]. In a recent study, Piitulainen and co-workers [57] introduced an MEG-compatible pneumatic hand/foot stimulator, which would be suitable for delivering the proprioceptive feedback. In further studies, we will investigate the effect of simultaneous MI and proprioceptive stimulation to mu rhythm modulation and decoding accuracy.

Second, in the current study we did not apply inverse modeling prior to feature extraction since our main goal was to examine whether sufficient decoding can be achieved with features extracted in sensor space and linearly-transformed subspaces. Operating in sensor space and SSD/CSP-transformed subspaces requires less computation and subject preparation than source-space analysis; for example, MRI scans prior to MEG measurements are not needed. It has been argued by Blankertz and colleagues [24] that CSP is an optimal method for finding variance differences in a given dataset, and inverse modeling does not bring any additional information to that problem. However, several studies have shown that source-level information increases the separability of various MI tasks [17,58,59] and would thus be beneficial in BCI systems. In addition, source-level analysis would have the spatial specificity required for targeting specific brain regions. This issue might be especially critical when dealing with classification tasks with more than two classes, as CSP is often suboptimal for such classification. Source imaging has been successfully applied to several real-time neurofeedback experiments using EEG [58,60] and MEG [61–64], and used in an offline classification analysis [65]. The added value of source-space feature extraction and classification will be evaluated in our further studies.

Third, the experimental protocol of this study was synchronous, i.e. the timing of epochs was pre-determined and the subjects were instructed to follow the visual cues indicating MI and rest. In recent studies, also asynchronous approaches for MI-BCI feedback have been proposed [66,67], in which the subject is allowed to perform MI at a self-determined pace. In this case, the data analysis should be done in sliding time windows over the course of the measurement, which requires more computation than processing one epoch at a time. In the current study, feature extraction and classification were done only during the discrete MI epochs and the feedback was shown immediately after the epoch. In the asynchronous feedback paradigm, the output of the classifier should be updated continuously, preferably with a minimal delay. Although this approach might be computationally more challenging, the asynchronous paradigm would be more realistic and thus give the user a stronger feeling of control over the neurofeedback system.

## 5. Conclusions

We compared different feature extraction methods for classifying MI-related MEG signals measured from healthy, untrained subjects. Spatial filtering with combined SSD and CSP outperformed other methods in discriminating left- vs. right-hand MI and rest from MI. Other spatial filtering methods yielded good accuracies as well, and CSP filtering also improved the discriminability of all time—frequency features. The level of 20-Hz suppression during MI correlated with the subjective MI-vs-rest classification accuracy, implying the potential use of 20-Hz suppression as a pre-screening tool before BCI training.
